# Clinical efficacy and its influencing factors of surgical treatment for T-shaped associated with posterior wall acetabular fractures using combined surgical approaches

**DOI:** 10.1186/s12893-022-01467-5

**Published:** 2022-02-23

**Authors:** Yun Yang, Chang Zou, Yue Fang, Sujan Shakya

**Affiliations:** Department of Orthopaedics, West China Hospital, Sichuan University, Chengdu, Sichuan People’s Republic of China

**Keywords:** Acetabular fractures, T-shaped, Posterior wall, Surgical approach, Surgical timing, Internal fixation

## Abstract

**Background:**

The objective of this study was to evaluate the outcomes of surgical treatment for T-shaped associated with posterior wall acetabular fractures using combined surgical approaches and its influencing factors.

**Methods:**

Between January 2009 and June 2018, a total of 21 patients with T-shaped acetabular fractures involving posterior wall were treated with combined approaches. The combined approaches were a combination of the Kocher-Langenbeck (KL) approach and the anterior approach (Stoppa or Ilioinguinal). The acetabular fractures in this study were divided into two groups respectively according to surgical approach and surgical timing: KL + Ilioinguinal (IL) approaches and KL + Stoppa approaches, early surgery and late surgery.

**Results:**

13 cases were treated within 14 days of injury. 15 cases were treated using the KL + Stoppa approaches and remaining 6 cases were treated using the KL + IL approaches. Anatomical and imperfect reduction were achieved in 12 cases (57.1%) with excellent to good clinical outcome in 42.9% of cases. Early surgery had a statistically significant improvement over late surgery in terms of quality of reduction and clinical outcomes. In the early surgery, the incidence of preoperative chest and abdomen injuries and postoperative deep vein thrombosis was significantly lower than that of the late surgery. There was no statistical difference between the KL + IL approaches and KL + Stoppa approaches in the demographics, preoperative associated injuries, quality of reduction, clinical outcomes and postoperative complications.

**Conclusions:**

The results of this study indicate that T-shaped associated with posterior wall acetabular fractures are difficult to treat surgically. Early surgery can improve the quality of fracture reduction, promote the recovery of hip function, and decrease the incidence of postoperative deep vein thrombosis. The main factor that affects surgical timing is the presence of preoperative chest and abdominal injuries. Compared with the KL + IL approaches, the KL combined with Stoppa approach can not significantly improve the clinical outcomes of such acetabular fractures.

## Background

shape acetabular fractures belong to complex fractures according to Judet-Letournel classification system [1]. The “T” shaped fractures of the hemipelvis are formed by a transverse fracture line and a vertical line through the obturator foramen. The current established principle is anatomical reduction and firm internal fixation to achieve a good outcome [2, 3].

Multiple factors that affect clinical outcomes include pre-existing associated injuries, surgical considerations, and postoperative complications [4]. The quality of the articular reduction has been shown to be the most important determinant of clinical outcomes. Recent reports found that the presence of a posterior wall fracture is an independent negative predictor for T-shaped acetabular fractures [5]. However, there is little literature that describes the surgical treatment of T-shaped acetabular fractures involving the posterior wall.

The surgical approach for acetabular fractures is crucial to achieving the goal of anatomic reduction of fractures with a minimum of complications [6]. For T-shaped fractures, common surgical approaches include a single anterior approach, a single posterior approach [7], and combined approaches if necessary. However, there is no consensus about the ideal approach. The most appropriate surgical approach becomes challenging when acetabular fracture involves both columns [8, 9]. When T-shaped fractures involve the posterior wall, alternative surgical approaches include a simple posterior approach and combined approaches.

In recent years, we have treated a number of T-shaped associated with posterior wall acetabular fractures through the KL approach combined with Stoppa or IL approach. Therefore, we retrospectively reported postoperative radiological results for a group of patients with such acetabular fractures, and evaluated the outcomes of surgical treatment using combined surgical approaches and its influencing factors.

## Materials and methods

### Subjects

A retrospective evaluation was conducted of patients with a T-shaped fracture involving posterior wall in a level-I trauma center between January 2009 and June 2018, with a minimum 12-month follow-up period. Inclusion criteria were as following: (1) age greater than or equal to 18 years; (2) radiological diagnosis of T-shaped associated with posterior wall acetabular fractures; (3) fractures treated with combined approaches. Exclusion criteria included open fractures, pathological fractures, and previous history of hip injuries. Additionally, those patients were excluded, who were lost in follow-up, and who suffered femoral head fracture. Data collected by medical records included demographic data, mechanism of injury, associated injuries, surgical timing, surgical approaches, and documented complications*.* The study complied with the Declaration of Helsinki and was approved by the ethics committee of West China Hospital. All patients provided written informed consent.

### Preoperative planning

At our unit, we primarily treated all patients with acetabular fractures according to Advanced Trauma Life Support (ATLS) protocol. Immediate reduction was done in cases with an associated hip dislocation. Ipsilateral skeletal traction was applied until surgery. Indication for internal fixation an acetabular fracture with 2 mm or more of displacement in the dome of the acetabulum [10].

### Surgical timing

Patients were divided into two groups according to surgical timing: early surgery and late surgery. Surgery within 14 days was defined as early surgery, and surgery beyond 14 days was defined as late surgery. All patients underwent surgery as soon as their general condition permitted. All surgeries were performed by the same surgical team.

### Surgical approach

**Fig. 1:**
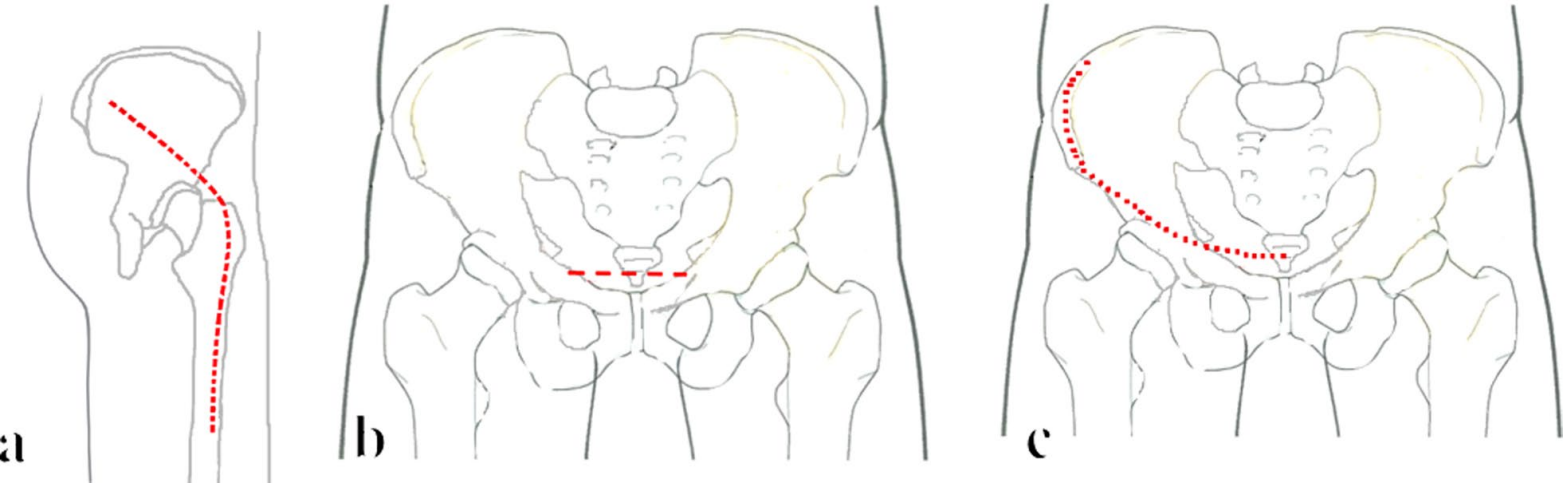
Incisions of three surgical approaches (**a**–**c** respectively represented KL approach, Stoppa approach and IL approach)

**Fig. 2:**
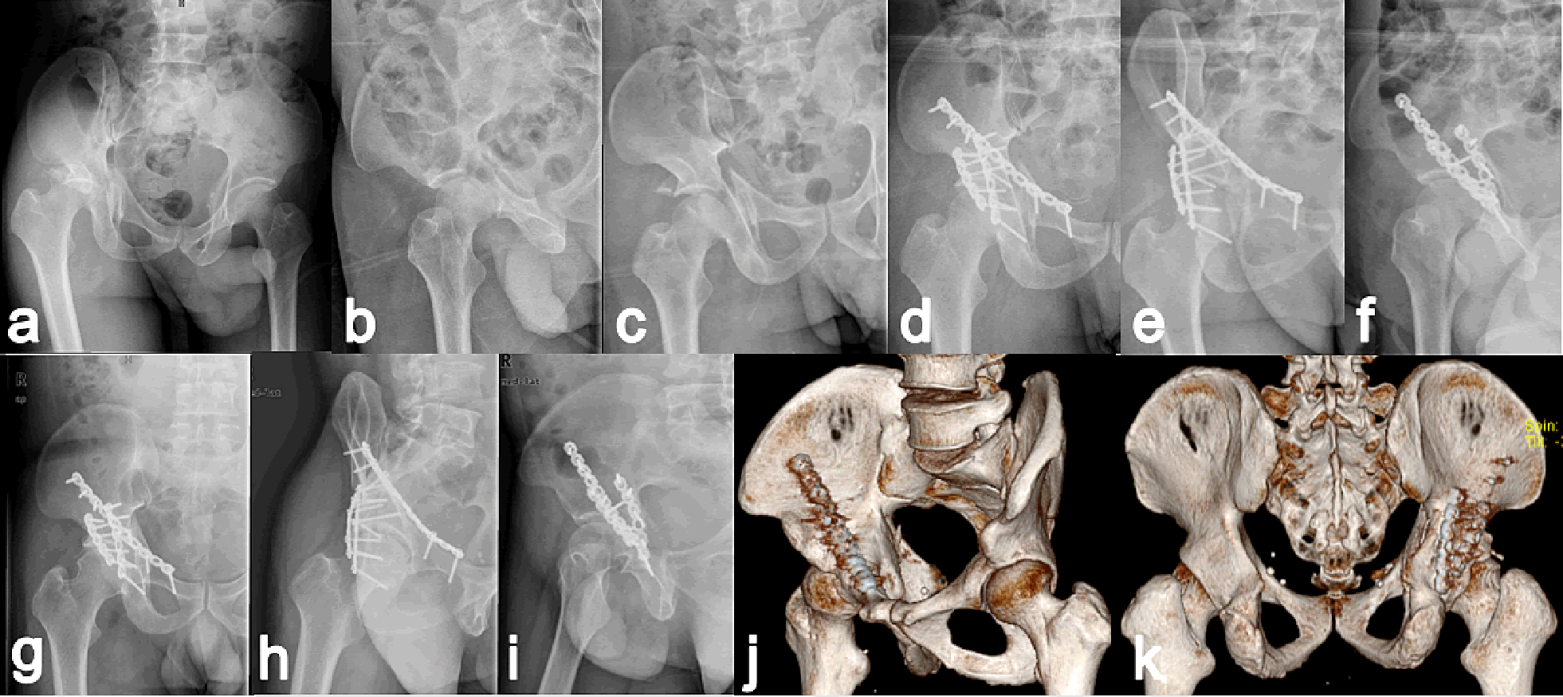
A 43-year-old male treated with KL + IL approaches 5 days after crush injury to hip. **a**–**c** Preoperative radiographs revealed T-shaped acetabular fracture involving the posterior wall; **d**–**f** Immediate postoperative radiographs showing imperfect reduction; **g**–**k** At 9 years of follow up, the patient presented good outcome with slight discomfort of right hip due to mild osteoarthritis, which verified by postoperative images

**Fig. 3:**
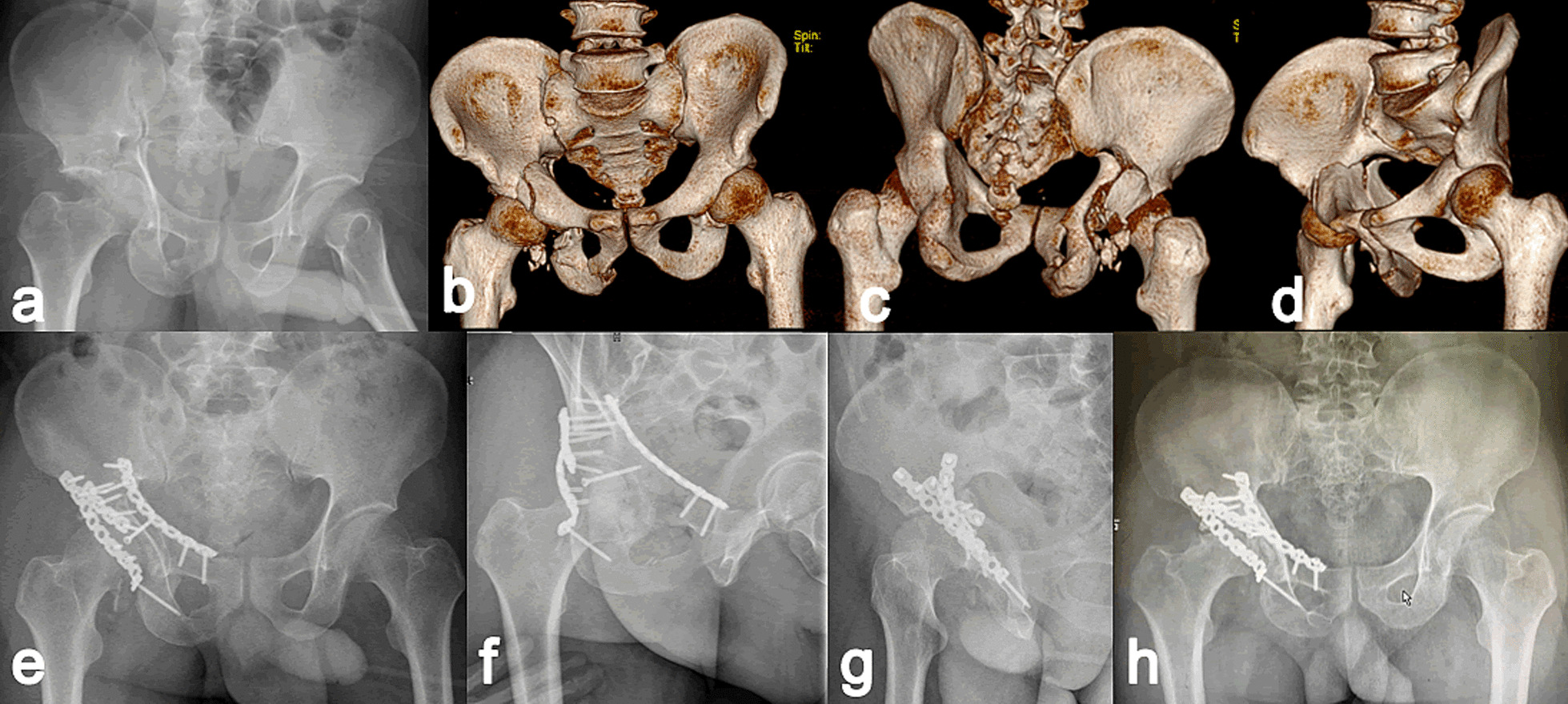
A 53-year-old male who sustained T-shaped acetabular fracture with the posterior wall treated with KL + Stoppa approaches 18 days after the traffic accident. **a** Preoperative anteroposterior radiograph; **b**–**d** Preoperative three-dimensional reconstructions; **e**–**g** Immediate postoperative radiographs showing imperfect reduction; **h** At 1 year of follow-up, the patient presented poor outcome with right hip pain and claudication due to avascular necrosis of the femoral head, which verified by postoperative radiograph

These cases were divided into two groups according to surgical approach: KL + IL (Figs. 1, 2) and KL + Stoppa (Figs. 1, 3). These patients were in the "floating" position. For KL approach, the distal part of the incision started 10–15 cm distal to the tip of the greater trochanter and extended to the greater trochanter along the longitudinal axis of the femur. The proximal part of the incision was extended from the tip of the greater trochanter to the posterior superior iliac spine (PSIS) to 6 cm from PSIS (Fig. 1a). The deep exposure of KL approach was done following the steps described by Letournel [11]. Separation of the quadratus femoris muscle should be avoided in order to protect the medial circumflex vessels. Be careful to protect the sciatic nerve at all times during the procedure. The position of knee bend and hip extension during surgery can help to reduce the risk of iatrogenic nerve injury.

Because the Stoppa approach can be adopted to treat all fractures that can be managed through the IL approach [12–14], we chose the Stoppa approach or IL approach as the anterior approach. The incision of the Stoppa approach was located 1 ~ 2 cm above the pubic symphysis (Fig. 1b). The rectus abdominis was dissected and retracted to expose the pubic symphysis and superior ramus of the pubis. If corona mortis were found, ligation should be performed. The iliopubic fascia and obturator fascia were incised to expose the true pelvic rim, the quadrilateral surface and the posterior column. The iliac crest approach can be adopted as an additional incision when the Stoppa approach cannot be adequately reduced fractures.

The incision of the IL approach started from the middle of the iliac crest and extended forward and distally through the anterior superior iliac spine (ASIS) to 2 cm above the pubis symphysis (Fig. 1c). Deep exposure could refer to the steps Tosounidis et al. Described [15]. There were four surgical windows, namely lateral, middle, medial and median Windows. In the surgical process, the above windows should be flexibly combined; During exposure, continuous catheterization to keep the bladder free of tension would help reduce the chance of its injury.

All fractures were fixed with reconstruction plate. Articular impaction if present was elevated and grafted by cancellous bone. The wound was closed over a drain.

### Postoperative care and follow-up

The postoperative protocol entailed non-weight bearing for 4 weeks. Protected weight bearing was gradually initiated at 8–12 weeks. Full weight bearing was allowed after 12 weeks. The radiographic follow-up was performed at 1, 3, 6 and 12 months. Thereafter, patients were examined at 1-year intervals. Displacement of 0 to 1 mm was considered as anatomical reduction according to the criteria described by Matta [16]. Two experienced surgeons independently performed radiographic measurements. Clinical outcomes were classified as excellent, good, fair, and poor according to the Matta modification of the Merle D’Aubigne score [16]. The minimum follow-up period was set at 1 year. All patients did attend the final follow-up.

### Statistical analysis

Statistical analysis of the data was performed using SPSS version 20.0 statistical software (SPSS Inc., Chicago, Illinois, USA). The results were presented as the mean ± standard deviation. Student’s t test was used for quantitative variables. Categorical variables were analyzed by Pearson’s Chi-square test or Fisher’s exact test where appropriate. The level of significance was set at a P value < 0.05.

## Results

There were 21 cases included in the study, with an average age of 43 years. All patients were followed up for an average of 47 months (range 12–108 months). The most common mechanism of injury was motor vehicle collision. 13 cases were treated within 14 days of injury. 15 cases were treated using the KL + Stoppa approaches and remaining 6 cases were treated using the KL + IL approaches. Five cases obtained anatomic reduction and 7 cases got imperfect reduction. By the end of follow-up, there were 9 patients with excellent to good outcomes (Table 1).

**Table 1:** The demographics, radiological and clinical outcomes of subjects

Variable	Value	Percent
Mean age (years)	43 (21–72)	–
Gender		
Male	16	76.2
Female	5	23.8
Side of injury		
Right	12	57.1
Left	9	42.9
Mechanism of injury		
Motor vehicle collision	13	61.9
Fall from height	5	23.8
Others	3	14.3
Surgical timing		
Early surgery	13	61.9
Late surgery	8	38.1
Surgical approach		
KL + IL	6	28.6
KL + Stoppa	15	71.4
Quality of reduction (mm)		
Anatomic (0–1)	5	23.8
Imperfect (2–3)	7	33.3
Poor (> 3)	9	42.9
Clinical outcome		
Excellent	3	14.3
Good	6	28.6
Fair	2	9.5
Poor	10	47.6

### Surgical timing

In terms of quality of reduction and clinical outcomes, early surgery had a statistically significant improvement over late surgery. There were no statistical differences in demographics between the two groups (Table 2). In the early surgery, the incidence of preoperative chest and abdomen injuries was significantly lower than that of the late surgery. Early surgery had a statistically significant decrease over late surgery in deep vein thrombosis (DVT). No cases of pulmonary embolism were noted. In addition, there was no significant difference in other complications (excluding DVT) between the two groups (Table 3). No intraoperative lesions of the sciatic nerve and major blood loss occurred in all patients.

**Table 2:** Comparison of demographics, radiological and clinical outcomes between the two groups

Variable	Early surgery	Late surgery	χ^2^	P value
Age (years)	45.1 ± 13.8	40.6 ± 9.6	–	0.427
Gender				
Male	10	6	0.010	0.920
Female	3	2		
Mechanism of injury				
Motor vehicle collision	7	6	2.212	0.331
Fall from height	3	2		
Others	3	0		
Surgical approach				
KL + IL	4	2	0.081	0.776
KL + Stoppa	9	6		
Quality of reduction (mm)				
Anatomic (0–1)	5	0	6.462	0.040
Imperfect (2–3)	5	2		
Poor (> 3)	3	6		
Clinical outcome				
Excellent	3	0	8.562	0.036
Good	5	1		
Fair	2	0		
Poor	3	7

**Table 3:** Comparison of associated injuries and postoperative complications between the two groups

Variable	Early surgery	Late surgery	χ^2^	P value
Associated injuries				
Chest, Abdominal	1	5	4.774	0.029
Limb/Spine fracture	4	3	0.028	0.867
Other injuries	7	2	3.316	0.069
Complications				
Wound infection	1	0	1.213	0.271
Deep vein thrombosis	1	6	3.914	0.048
Post-traumatic arthritis	3	3	0.046	0.829
Heterotopic ossification	4	3	0.465	0.495
Dislocation of hip	1	1	0.013	0.910
Avascular necrosis	2	1	0.574	0.449

### Surgical approach

There was no significant difference between the groups in demographics. There was also no statistically significant difference when the quality of reduction and clinical outcomes were compared between the KL + IL approaches and KL + Stoppa approaches (Table 4). Comparing associated injuries and postoperative complications, there was no significant difference between the groups (Table 5).

**Table 4:** Comparison of demographics, radiological and clinical outcomes between the two groups

Variable	KL + IL	KL + Stoppa	χ^2^	P value
Age (years)	40.5 ± 9.5	44.6 ± 13.4	–	0.505
Gender				
Male	5	11	0.236	0.627
Female	1	4		
Mechanism of injury				
Motor vehicle collision	4	9	0.244	0.885
Fall from height	1	4		
Others	1	2		
Surgical timing				
Early surgery	4	9	0.081	0.776
Late surgery	2	6		
Quality of reduction (mm)				
Anatomic (0–1)	1	4	0.280	0.869
Imperfect (2–3)	2	5		
Poor (> 3)	3	6		
Clinical outcome				
Excellent	1	2	0.910	0.823
Good	2	4		
Fair	0	2		
Poor	3	7

**Table 5:** Comparison of associated injuries and postoperative complications between the two groups

Variable	KL + IL	KL + Stoppa	χ^2^	P value
Associated injuries				
Chest, Abdominal	3	6	0.151	0.697
Limb/Spine fracture	2	3	0.010	0.920
Other injuries	3	4	0.101	0.751
Complications				
Wound infection	0	1	0.714	0.398
Deep vein thrombosis	4	3	0.398	0.305
Post-traumatic arthritis	2	5	0.580	0.446
Heterotopic ossification	3	4	0.018	0.895
Dislocation of hip	0	2	1.485	0.223
Avascular necrosis	2	1	0.940	0.332

## Discussion

For T-shaped acetabular fractures involving the posterior wall, the ischio-pubic ramus segment is free-floating. In addition to restoring the roof and the posterior wall, rotation of this segment can be difficult to reduce. Our results showed T-shaped associated with posterior wall acetabular fractures were difficult to treat surgically and had poor prognosis. The findings were confirmed in the results reported by others [16, 17]. Of the 14 fractures of this type reported by Matta [16], only 8 patients achieved excellent and good outcomes, despite relatively high rate of anatomical reduction. Briffa et al. [17] identified the T-shaped fracture with an associated posterior wall fracture as the “worst case scenario” as this fracture is both difficult to reduce.

As all columns are often displaced and rotated, the choice of surgical approaches for the T-shaped acetabular fractures has been a considerable challenge over recent decades [18–22]. Still, there are some rules to refer. The level of the transverse element is the most important factor in the choice of surgical approach, as is the case for simple transverse fracture. The choice of initial surgical approach depends on the larger displacement and the higher level of of the transverse fracture. The KL approach is the most commonly used due to the obvious posterior displacement of most T-shaped fractures. If the transverse portion is high and the posterior column is displaced, greater trochanteric osteotomy and dislocation of the hip can be performed to achieve reduction of the posterior column, so that the articular surface can be observed simultaneously. In addition, after assessing column displacement and the level of the transverse element, attention should be paid to the vertical fracture line. If the vertical fracture line passes through the ischium, the posterior approach must be used. If the vertical fracture line passes through the obturator foramen and the fracture characteristics meet the criteria for the anterior approach, the anterior approach may be used. Gusic et al. [23] recommend a single KL approach in all T-shaped fractures apart from T-shaped associated with the anterior wall fracture. Of course, manipulation of the posterior column fracture fragement through the posterior approach to control the separated anterior column fracture fragement often requires a combination of several techniques for reduction. These techniques include: Schanz needle for femoral traction or ischial rotation control, Jungbluth forceps for reduction of the posterior column, pelvic plates for maintenance reduction or temporary fixation, and the use of lag screws, etc. Letornel considered the use of Farabeuf forceps and two screws to prioritize reduction of the posterior column [1]. For concomitant posterior wall fractures, special reduction tools such as Picador forceps and point-type reduction forceps are also required. Compression fractures along the joint rim should be treated prior to reduction of the posterior wall fractures.

If indirect reduction of the anterior column cannot be obtained through the posterior approach, additional anterior approach need to be considered. When placing temporary or final implants, the internal implants should be avoided by crossing from one column to another to prevent the reduction of the opposite column. The anterior column can be first reduced to the residual acetabular crest above the ilium. During this process, instruments such as Faraboeuf forceps, Schanz screws, pelvic reduction forceps or bone splitters can be used to aid reduction. Moreover, Kreder et al. [24] used the KL approach to treat posterior wall component of a complex acetabular fracture and used a sequential IL approach in selected cases of T-shaped. For T-shaped fractures, they had 18% poor reductions in 11 cases. Harris et al. [18] used simultaneous iliofemoral and KL approach for 10 T-shaped acetabular fractures and had overall 71% anatomical, 21% imperfect and 8% poor reductions. In our study, all fractures were fixed by the combined approaches (KL + Stoppa or KL + IL). There was no statistically significant difference in the terms of the quality of reduction and clinical outcomes between the both groups. There were overall 24% anatomical, 33% imperfect and 43% poor reductions.

Although the clinical outcomes of open reduction and internal fixation (ORIF) have been related to surgical timing, the ideal timing for acetabular fracture surgery is controversial. Several authors advocate acute intervention [25, 26], while others suggest that early surgical intervention can result in increased blood loss [27]. Patients with acetabular fractures often have multiple injuries, which often require emergent management. These patients are deemed unfit for early surgical intervention. Early surgical treatment within 14 days of injury is more successful, while delayed surgery of more than 2 weeks tends to decrease the quality of reduction and could hamper the ultimate outcomes [16, 28, 29]. In the current study, most patients with early surgery were operated on within 5–7 days of injury. The main factor that affected surgical timing was the presence of associated injuries, especially the chest or abdominal injuries. The results showed early surgery could improve the quality of reduction and the clinical outcomes, and could reduce the incidence of DVT.

This study had several limitations. Firstly, the study was prone to various forms of bias due to its retrospective nature. Secondly, the cohorts of early and late surgeries were not equal: a smaller number of patients were treated 14 days after the injury. Finally, the study was of small size and a relatively short follow-up duration.

## Conclusion

Based on the results of our study, we concluded that T-shaped associated with posterior wall fractures have poor prognosis. Compared with the KL + IL approaches, the KL combined with Stoppa approach can not significantly improve the clinical outcomes. Early surgery can improve the quality of reduction and decrease the incidence of postoperative deep vein thrombosis. The main factor that affects surgical timing is the presence of preoperative chest and abdominal injuries. In the future, long-term, prospective and randomized studies are warranted to verify the validity and accuracy of this study.

## Data Availability

Datasets are available from the corresponding author on reasonable request.

## Abbreviations

KL: Kocher–Langenbeck
IL: Ilioinguinal
ATLS: Advanced trauma life support
PSIS: Posterior superior iliac spine
ASIS: Anterior superior iliac spine
DVT: Deep vein thrombosis
ORIF: Open reduction and internal fixation
